# Diagnostic Accuracy of Contemporary Selection Criteria in Prostate Cancer Patients Eligible for Active Surveillance: A Bayesian Network Meta-Analysis

**DOI:** 10.3389/fonc.2021.810736

**Published:** 2022-01-10

**Authors:** Yu Fan, Yelin Mulati, Lingyun Zhai, Yuke Chen, Yu Wang, Juefei Feng, Wei Yu, Qian Zhang

**Affiliations:** ^1^ Department of Urology, Peking University First Hospital, Beijing, China; ^2^ Institute of Urology, Peking University, Beijing, China; ^3^ National Urological Cancer Center, Beijing, China; ^4^ Department of Urology, Tibet Autonomous Region People’s Hospital, Lhasa, China; ^5^ Department of Urology, The Second Affiliated Hospital of Chongqing Medical University, Chongqing, China; ^6^ Department of Surgery, Khoo Teck Puat Hospital, Singapore, Singapore; ^7^ Peking University Binhai Hospital, Tianjin, China

**Keywords:** prostate cancer, active surveillance, selection criteria, diagnostic accuracy, Epstein criteria, network meta-analysis

## Abstract

**Background:**

Several active surveillance (AS) criteria have been established to screen insignificant prostate cancer (insigPCa, defined as organ confined, low grade and small volume tumors confirmed by postoperative pathology). However, their comparative diagnostic performance varies. The aim of this study was to compare the diagnostic accuracy of contemporary AS criteria and validate the absolute diagnostic odds ratio (DOR) of optimal AS criteria.

**Methods:**

First, we searched Pubmed and performed a Bayesian network meta-analysis (NMA) to compare the diagnostic accuracy of contemporary AS criteria and obtained a relative ranking. Then, we searched Pubmed again to perform another meta-analysis to validate the absolute DOR of the top-ranked AS criteria derived from the NMA with two endpoints: insigPCa and favorable disease (defined as organ confined, low grade tumors). Subgroup and meta-regression analyses were conducted to identify any potential heterogeneity in the results. Publication bias was evaluated.

**Results:**

Seven eligible retrospective studies with 3,336 participants were identified for the NMA. The diagnostic accuracy of AS criteria ranked from best to worst, was as follows: Epstein Criteria (EC), Yonsei criteria, Prostate Cancer Research International: Active Surveillance (PRIAS), University of Miami (UM), University of California-San Francisco (UCSF), Memorial Sloan-Kettering Cancer Center (MSKCC), and University of Toronto (UT). I^2^ = 50.5%, and sensitivity analysis with different insigPCa definitions supported the robustness of the results. In the subsequent meta-analysis of DOR of EC, insigPCa and favorable disease were identified as endpoints in ten and twenty-two studies, respectively. The pooled DOR for insigPCa and favorable disease were 0.44 (95%CI, 0.31–0.58) and 0.66 (95%CI, 0.61–0.71), respectively. According to a subgroup analysis, the DOR for favorable disease was significantly higher in US institutions than that in other regions. No significant heterogeneity or evidence of publication bias was identified.

**Conclusions:**

Among the seven AS criteria evaluated in this study, EC was optimal for positively identifying insigPCa patients. The pooled diagnostic accuracy of EC was 0.44 for insigPCa and 0.66 when a more liberal endpoint, favorable disease, was used.

**Systematic Review Registration:**

[https://www.crd.york.ac.uk/prospero/], PROSPERO [CRD42020157048].

## Introduction

An estimated 1.28 million new cases of prostate cancer (PCa) occurred in 2018 worldwide (1), and PCa remains the second most commonly diagnosed cancer in men (2). PCa has an indolent natural history in most cases, and most patients die of other causes before disease progression (3). Due to the widespread use of prostate-specific antigen (PSA) screening, many of these cancers are detected when they are in the early stage, low-grade, and localized (4).

With the intention of avoiding overtreatment and preserving quality of life, active surveillance (AS) was originally suggested in 1994 (5), Epstein et al. first introduced the definition of clinically insignificant prostate cancer (insigPCa), which is defined as organ-confined, no Gleason pattern 4/5 and small volume PCa, and the Epstein criteria (EC) was established to predict these insigPCa. Since then, AS has been offered as an alternative to immediate curative intervention in men with favorable-risk PCa. Most patients are monitored on surveillance with PSA and digital rectal examination (DRE) at least biannually, and received surveillance prostate biopsies at a 1–2-year interval. Interventions were taken once high-grade disease was found on surveillance biopsies. The 15-year disease-specific mortality rate of AS is lower than 5% in men with low-risk PCa (6), and AS leads to a better quality-adjusted life experience than is reported by those who undergo curative treatment (7). Consequently, the population considered suitable for AS has rapidly expanded in recent years. The National Comprehensive Cancer Network (NCCN) now recommends AS as the preferred management option for men with very low-risk and low-risk PCa with over a 20-year and 10-year expected survival, respectively, and suggests that AS can even be considered in patients with favorable intermediate-risk cancer (8).

Several eligibility criteria have been established for AS based on published findings from large cohort studies. These criteria include clinical stage, PSA level, PSA density (PSAD = PSA level/prostate volume), Gleason score (GS), number of positive cores, and maximum cancer involved of a single core. However, the eligibility characteristics used to screen patients vary widely across different institutions, and there is currently no consensus on which criteria are optimal (9).

The misclassification rates of AS criteria are controversial. Some research has indicated that AS selection criteria may underestimate disease grade and extent in a small number of cases (10). However, in studies that evaluated upgrading in patients who underwent radical prostatectomy (RP), approximately 30% of men with a Gleason score of 5–6 based on needle biopsy were found to have higher-grade disease during RP (10–12). Meanwhile, several widely-used AS programs noted approximately the same upgrading rate on their first repeat biopsy within 1 year of diagnosis (13–15). These similarities strongly suggest that initial misclassification is the most common reason for reclassification at first-year surveillance biopsy (14, 16).

Variation in the AS selection criteria may result in different diagnostic accuracies (17). As far as we know, no direct comparison of large sample data has been done in this field yet. In this study, we used a Bayesian network meta-analysis (NMA) to indirectly compare the diagnostic accuracy of contemporary AS criteria and provide a diagnostic-accuracy ranking. Then, to further validate the absolute diagnostic odds ratio (DOR, i.e., accurately diagnosed rate) of top-ranked criteria derived from the NMA, another meta-analysis of DOR was performed.

## Methods

This study adhered to the recommendations of the Meta-Analyses of Observational Study in Epidemiology (MOOSE) group (18) and it was pre-registered in PROSPERO (with ID: CRD42020157048).

### Search Strategy

First, in order to identify the optimal AS criteria, we systematically reviewed PubMed for articles that were published from January 2008 to May 2019 for our NMA. The following search strategy was used: ((protocols [Title/Abstract]) OR criteria [Title/Abstract])) AND ((active surveillance [Title/Abstract]) AND prostatectomy [Title/Abstract]. Then, to further validate the DOR of the optimal AS criteria, we performed a second systematic search of PubMed articles published before March 2020 using the following search strategy: (((protocol [Title/Abstract]) OR criteria [Title/Abstract])) AND ((Epstein [Title/Abstract]) OR (Hopkins[Title/Abstract])OR (Insignificant[Title/Abstract])) AND prostatectomy[Title/Abstract]).

### Inclusion and Exclusion Criteria

The research strategy was framed by PICOS format. The two screening steps shared common inclusion and exclusion criteria. Each study was only included in the analysis if it met the following criteria: (1) the study was retrospective in design; (2) the participants fulfilled the requirements of any AS criteria and were treated with RP without neoadjuvant androgen deprivation treatment; (3) a head-to-head comparison of the diagnostic accuracies of two or more AS criteria was presented (note that this inclusion criterion was applied only to the NMA); and (4) postsurgical pathology (RP specimen) results were available, especially for cases of pathologically insignificant PCa (insigPCa) or favorable disease. Two definitions of insigPCa were applied: the classical definition (organ-confined Gleason score (GS) ≤6 (no Gleason pattern 4/5, i.e., International Society of Urological Pathology (ISUP) score = 1; and tumor volume <0.5 cm^3^) (19); and the updated definition (organ-confined GS ≤6; index and overall tumor volume <1.3 and <2.5 cm^3^, respectively) (20). Favorable disease was defined as organ-confined, GS ≤ 6 with a negative surgical margin. The following were defined as exclusion criteria: (1) published in a language other than English; (2) absence of data on insignificant cancer; and (3) reviews, meeting posters, comments, and study criteria. Two researchers independently reviewed the title and abstract of each included study to identify articles for full-text screening. A third author was consulted to resolve any disagreements.

### Data Extraction

A predesigned form was used to extract general information and postoperative pathology characteristics for analysis. The following summary data were recorded: first author, year of publication, year of study recruitment, region, total number of patients, mean age, mean preoperative PSA, mean number of biopsy cores, the AS criteria examined, and the number of patients eligible for each protocol, and also the number of insigPCa cases. Our main outcome was diagnostic odds ratio (DOR) = percentage of pathologically insigPCa or favorable disease accurately diagnosed by each criterion.

### Statistical Analyses

The network plot of the comparisons among the seven AS criteria was generated using STATA SE 15 software (21). Odds ratios (ORs) with 95% credibility intervals (Crls) were used as summary characteristics to quantify the performance of each AS criterion relative to that of EC (recommended in the AUA\NCCN\EAU guideline) in the NMA. A forest plot was created to compare AS criteria with EC using a Bayesian model and Markov chain Monte Carlo methods in R 3.5.3 (22), random and fixed effects models were created to evaluate reported outcomes; a random effects model was selected if significant heterogeneity was identified. Surface under the cumulative ranking (SUCRA) analysis was then conducted to obtain a hierarchy of the seven AS criteria according to their relative performance (23). Forest plots of diagnostic accuracy were generated for each AS criteria to sketch profiles of the absolute DORs.

Publication bias was tested using funnel plots and Egger’s regression test (24), with asymmetrical, skewed and inverted funnels indicating the presence of publication bias (25). Heterogeneity was assessed using forest plots and I2 statistics. I2 values greater than 25, 50, or 75% indicate low, moderate, or high heterogeneity, respectively (26).

Sensitivity analysis was conducted to determine whether the applied definition of insigPCa (classical versus updated) affected the NMA results. First, the NMA included only studies that reported the use of both definitions, to enable us to validate the internal robustness of the results obtained when using each definition individually. Second, we analyzed studies that applied different combinations of insigPCa definitions: InsigPCa1 (6 studies with a classical definition & 1 with an updated definition) and InsigPCa2 (4 studies with a classical definition & 3 with an updated definition). The robustness of the NMA results was validated by comparing the forest plots and ranking plots obtained using different combinations.

A systematic meta-analysis of the DOR was performed for further validation of diagnostic accuracy with the criteria found to achieve the best rank in the SUCRA analysis. Forest plots were generated to estimate the pooled DOR of insigPCa and favorable disease. Heterogeneity was estimated; if significant heterogeneity was found, subgroup analysis and meta-regression were performed to evaluate the potential influencing factors.

## Results

### Network Meta-Analysis

**Figure 1:**
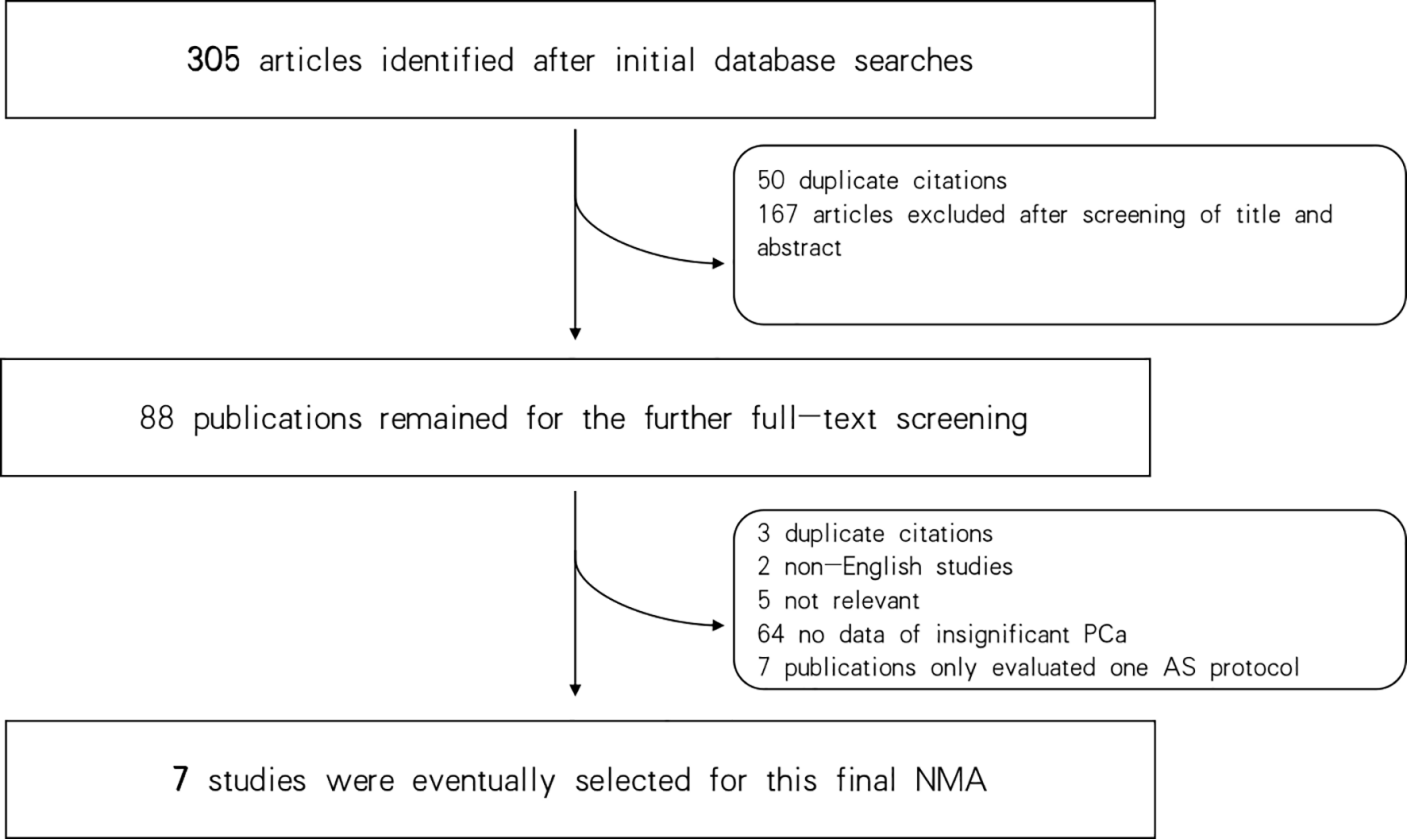
Flowchart of the study selection process and design.

Three hundred and five articles were identified in the initial database search. Of these, 50 duplicates were excluded, and 167 additional articles were excluded after reviewing their titles and abstracts. Consequently, 88 publications remained for full-text screening. Of these, 7 studies were selected for the final NMA (27–33). Seven criteria were finally identified in this study: EC (5), Prostate Cancer Research International: Active Surveillance (PRIAS) (34), Memorial Sloan-Kettering Cancer Center (MSKCC) (35), University of California, San Francisco (UCSF) (36), University of Miami (UM) (37), University of Toronto (UT) (38), and Yonsei criteria (31); for details of these included critera see **Supplementary Table 1**. **Figure 1** shows the flowchart of the selection procedure.

The baseline characteristics of the patients in the seven included studies are summarized in **Table 1**. All seven were retrospective studies published from January 2008 to May 2019. A total of 3,336 participants were included in this NMA. All these men accepted RP soon after the diagnosis through the AS criteria with TRUS guided biopsy. The EC and PRIAS criteria were analyzed in all 7 included studies (27–33), the MSKCC criteria was analyzed in 6 studies (28–33), the UCSF and UM criteria were analyzed in 5 studies (28–31, 33), the UT protocol was analyzed in 2 studies (30, 32), and the Yonsei protocol was analyzed in only one study, by Lim et al. (31). Four studies used the classical definition of insigPCa as a pathological endpoint (29–32), Iremashvili et al. (28) and Yamada et al. (33) applied both classical and updated definitions (28, 33), and Cantiello et al. used only an updated definition (27).

**Table 1:** Baseline characteristics of studies eligible for the network meta-analysis.

Study	Year	Region	Included AS protocols	No. of patients eligible to AS	Median age/years (median;IQR)	Median pre-operative PSA/ng/ml (median;IQR)	Mean No. of biopsy cores (mean; IQR)	Definition of insigPCa
Cantiello et al. (16)	2015	EU	PRIAS	188	66.0 (61.0–67.0)*	4.76 (4.05–7.01)*	NA (≥10)	Updated
			EC	96	65.0 (60.0–68.0)*	5.43 (4.26–7.08)*		
Iremashvili et al. (17)	2012	US	EC	109	60.8 (56.0–64.6)	5.0 (4.0–7.3)	11.3 (10–18)	Classical and update
			MSKCC	246				
			PRIAS	190				
			UCSF	270				
			UM	189				
Kang et al. (18)	2015	Asia	EC	70	62.0 (57.0–67.0)	5.4 (4.3–6.9)	NA(≥10)	Classical
			MSKCC	161				
			PRIAS	109				
			UCSF	141				
			UM	96				
Kim et al. (19)	2014	Asia	EC	137	66.0 (61.0–70.0)	5.5 (4.0–9.0)	NA (≥10)	Classical
			UT	387				
			UCSF	334				
			PRIAS	226				
			UM	222				
			MSKCC	322				
Lim et al. (20)	2013	Asia	EC	31	63.2 ± 7.7**	7.9 ± 0.3**	12.2 ± 1.8**	Classical
			MSKCC	121				
			PRIAS	101				
			UCSF	159				
			UM	88				
			Yonsei	69				
Palisaar et al. (21)	2012	EU	MSKCC	308	65.0 (42.0–77.0)	9.4 (0.6–83)	12.4 (10.0–32.0)	Classical
			EC	99				
			UT	514				
			PRIAS	174				
Yamada et al. (22)	2015	Asia	EC	35	67.0 (48–75)	6.0 (1.05–19.9)	NA	Classical and updated
			PRIAS	55				
			UM	69				
			UCSF	89				
			MSKCC	92				
			UT	118				

*Baseline data of each AS protocol; **Data provided with: mean + SD. IQR, inter quartile range; insigPCa, insignificant Prostate cancer; EC, Epstein Criteria; PRIAS, Prostate Cancer Research International: Active Surveillance; MSKCC, Memorial Sloan-Kettering Cancer Center; UCSF, University of California, San Francisco; UM, University of Miami; UT, University of Toronto.

**Figure 2:**
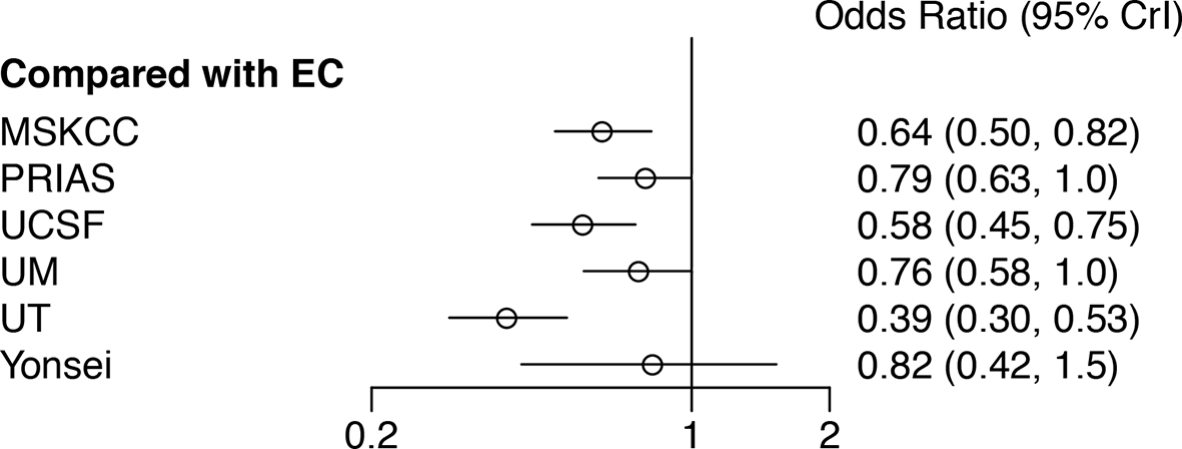
Forest plot showing a comparison of the diagnostic performance of all other included AS criteria in comparison to that of the EC. An OR greater than one represents a benefit relative to EC in terms of diagnostic accuracy for insignificant prostate cancer. EC, Epstein Criteria; PRIAS, Prostate Cancer Research International: Active Surveillance; MSKCC, Memorial Sloan-Kettering Cancer Center; UCSF, University of California, San Francisco; UM, University of Miami; UT, University of Toronto; Crl, Credible interval.

**Figure 3:**
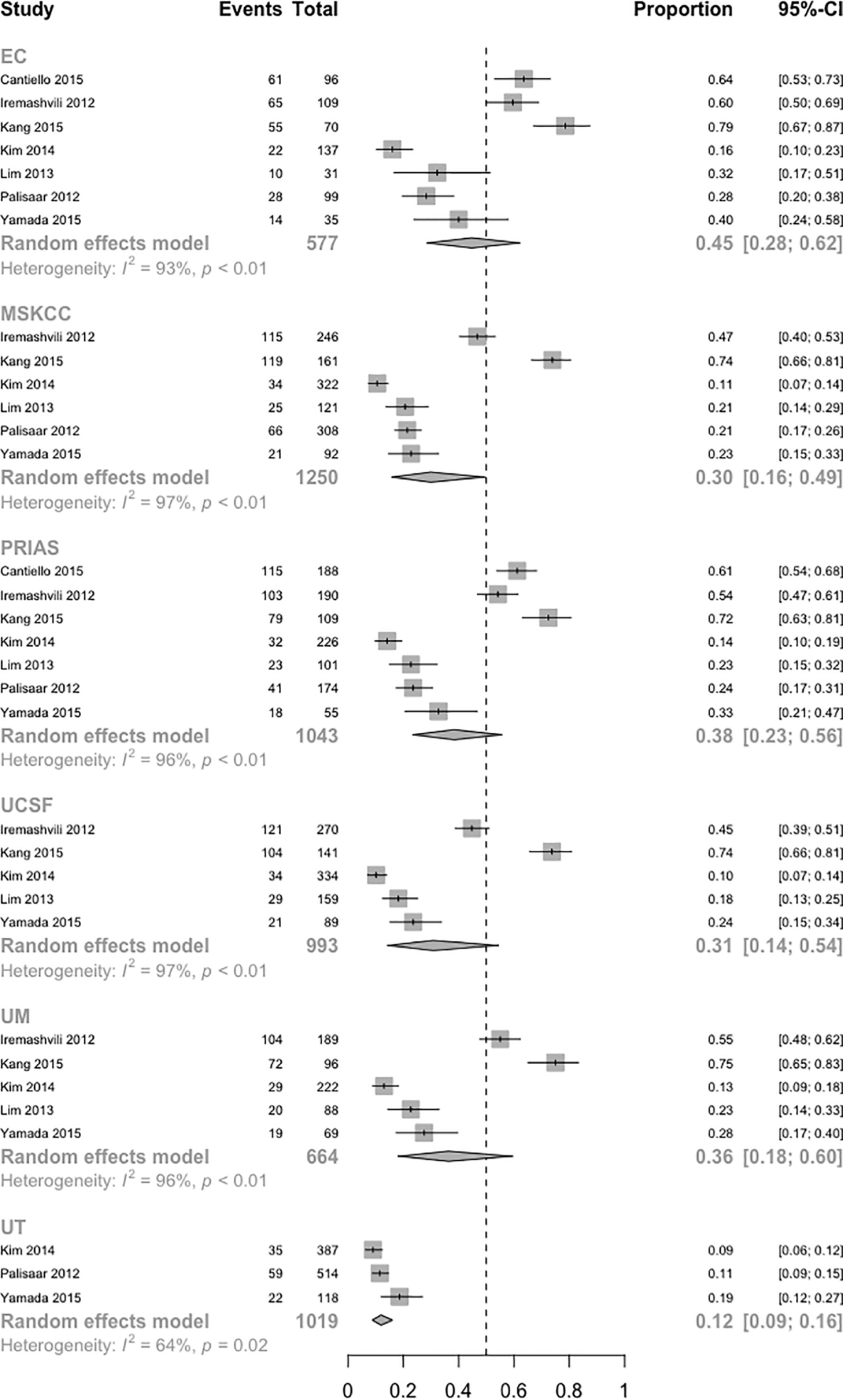
Population-weighted pooled diagnostic accuracy of each AS protocol. (InsigPCa1, including 6 studies with classical definition and 1 study with updated definition of insigPCa). Note: The absolute diagnostic accuracy of the Yonsei protocol was calculated based on only Lim et al, which was 0.25.

**Figure 4:**
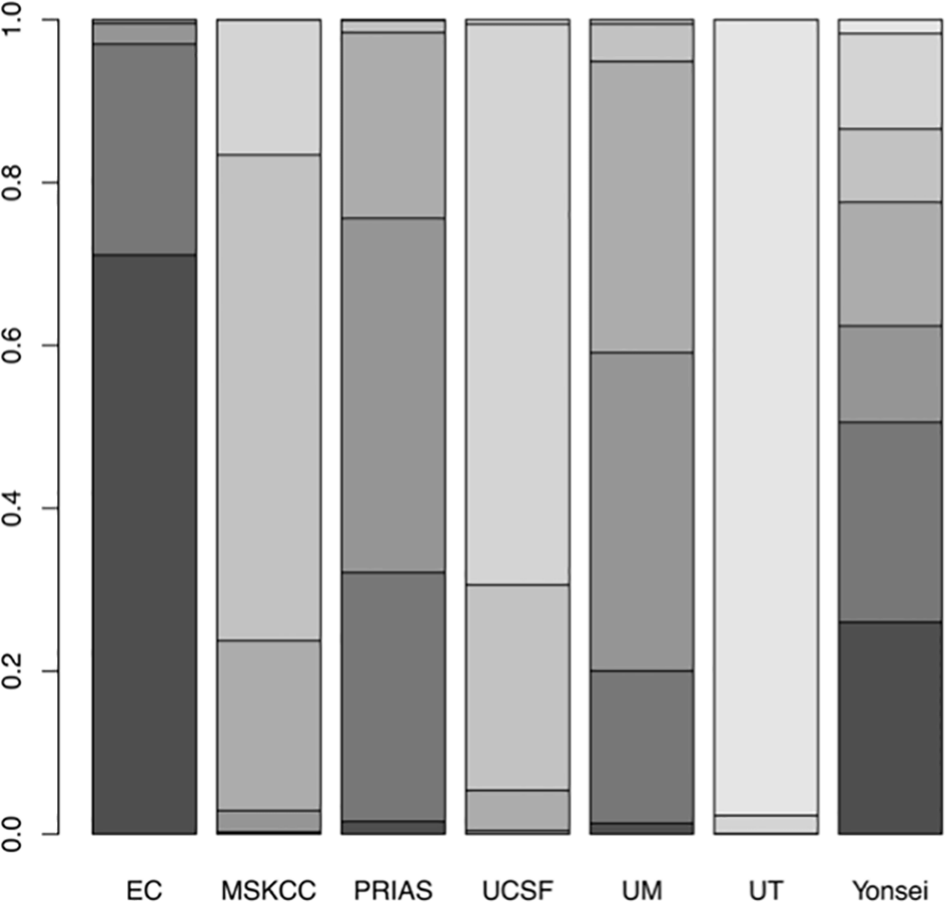
Surface under the cumulative ranking (SUCRA) plot of the 7 included AS protocols. A darker color is proportional to a better performance in predicting insigPCa.

A network plot was constructed to illustrate the comparisons of the seven AS criteria (**Supplementary Figure 1**). A forest plot showing the comparisons between each AS criteria and the EC is shown in **Figure 2**. Compared to all other criteria except for the Yonsei protocol, the EC was significantly better in predicting pathological insigPCa, and the pooled diagnostic accuracy of EC was 0.45 (95% Crl, 0.28–0.62) (see **Figure 3**). However, only one article reported the diagnostic accuracy of the Yonsei criteria (DOR = 0.25). Diagnostic accuracy of each AS criteria to identify patients with insigPCa is shown in detail in **Supplementary Table 2**. Because the Crl was wide, there was no significant difference between the EC and Yonsei criteria in their ability to predict insigPCa (OR, 0.82; 95% Crl, 0.42–1.50). The Yonsei protocol had no significant advantage over other AS criteria except for the UT protocol (OR, 0.48; Crl, 0.24–0.92) (**Supplementary Figure 2**). A SUCRA plot of these seven AS criteria is presented in **Figure 4**. When the seven AS criteria were ranked from best to worst according to their ability to positively predict insigPCa, their order was as follows: EC, Yonsei, PRIAS, UM, UCSF, MSKCC, and UT.

Moderate heterogeneity was found in the NMA (I^2^ = 50.5%, **Supplementary Figure 3**). There was no strong evidence of publication bias, and the funnel plot showed a certain degree of symmetry (**Supplementary Figure 4**).

Sensitivity analysis was carried out in two steps. First, to evaluate the two studies in which both the classical and updated definitions were used, the NMA was conducted on each definition respectively and the results consistently show that among the included criteria, EC was performed best (**Supplementary Figures 5, 6**). Second, the analysis of the InsigPCa1 and InsigPCa2 combinations again showed that EC was the optimal protocol (for InsigPCa1, see **Figure 2**; and for InsigPCa2, see **Supplementary Figure 7**), and the relative ranking of the criteria remained stable (for InsigPCa1, see **Figure 4**; and for InsigPCa2, see **Supplementary Figure 8**).

### Meta-Analysis of the DOR of the Optimal AS Criteria Derived From the NMA

After the initial database search, 163 articles were identified in a second search for studies that presented meta-analyses of the DOR achieved by the EC in either insigPCa or favorable disease. After reviewing the titles and abstracts of these articles, 117 were excluded, and 46 remained for further full-text screening. In all, 10 and 22 studies were selected for the meta-analyses of the DOR of the EC in insigPCa and favorable disease, respectively (for the flowchart of this study, see **Supplementary Figure 10**).

**Figure 5:**
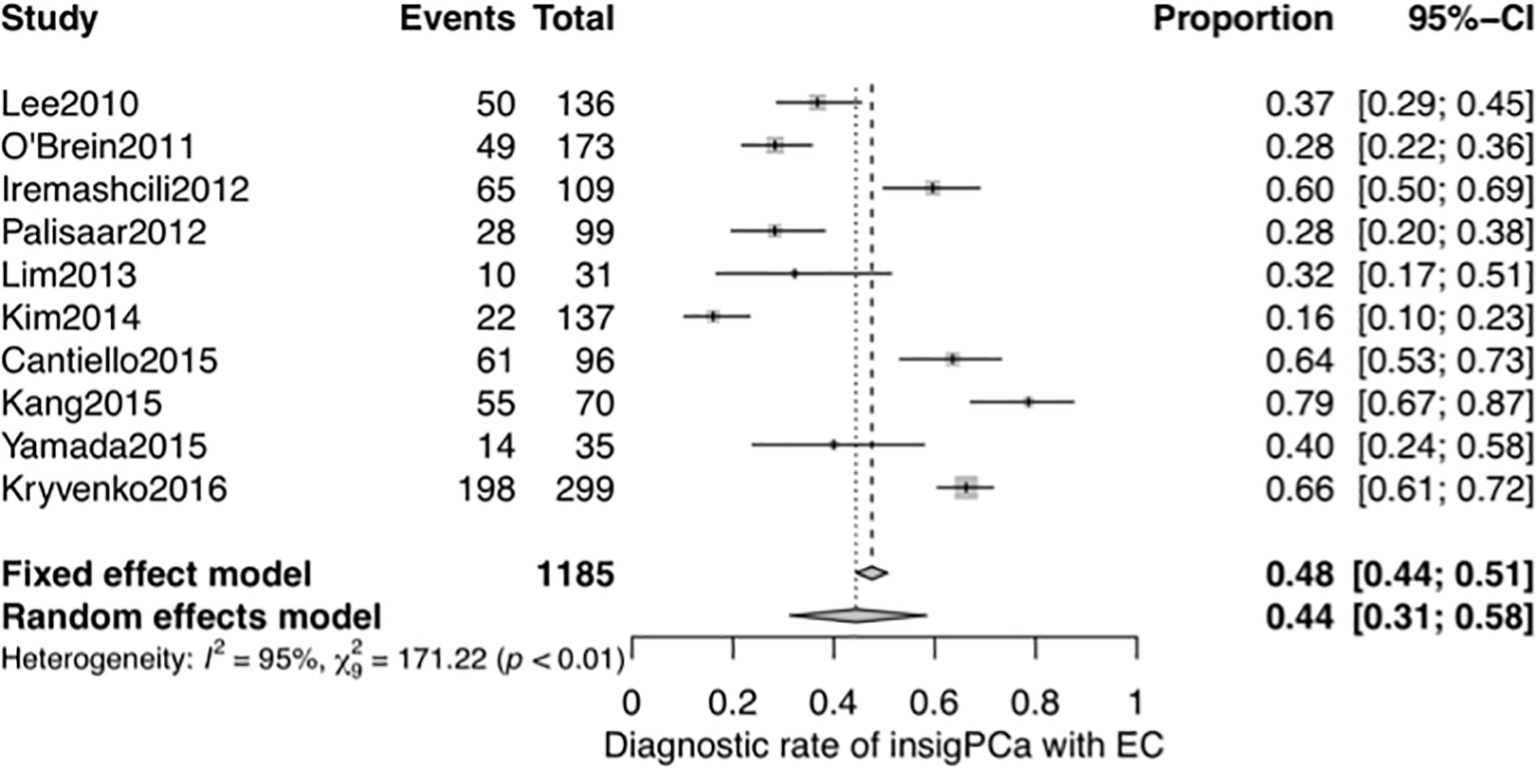
Forest plot of studies that explored the diagnostic accuracy of the EC for insigPCa.

A systematic meta-analysis was performed to validate the diagnostic accuracy of the EC for insigPCa. In all, 1,185 men were included from 10 studies (7 studies were same to the NMA with 3 additional studies) (27–33, 39–41), and the pooled DOR was 0.44 (95% Crl, 0.31–0.58, see **Figure 5**), consistent with results of the previously pooled analysis of the original 7 studies. While all 10 of these studies used the classical definition of insigPCa, there was significant heterogeneity (I^2^ = 95%). We considered that the region in which the studies were performed (inside or outside the US), the median duration of the study recruitment period, sample size and whether the central pathology was reviewed (yes or no) may represent potential sources of heterogeneity. While the subgroup analysis and meta-regression revealed no statistically significant differences for any of these factors (see **Supplementary Figures 11**, **12** and **Supplementary Table 3**). The funnel plot showed no asymmetry suggestive of publication bias (see **Supplementary Figure 13**), and the P-value of Egger’s regression test was 0.7427.

**Figure 6:**
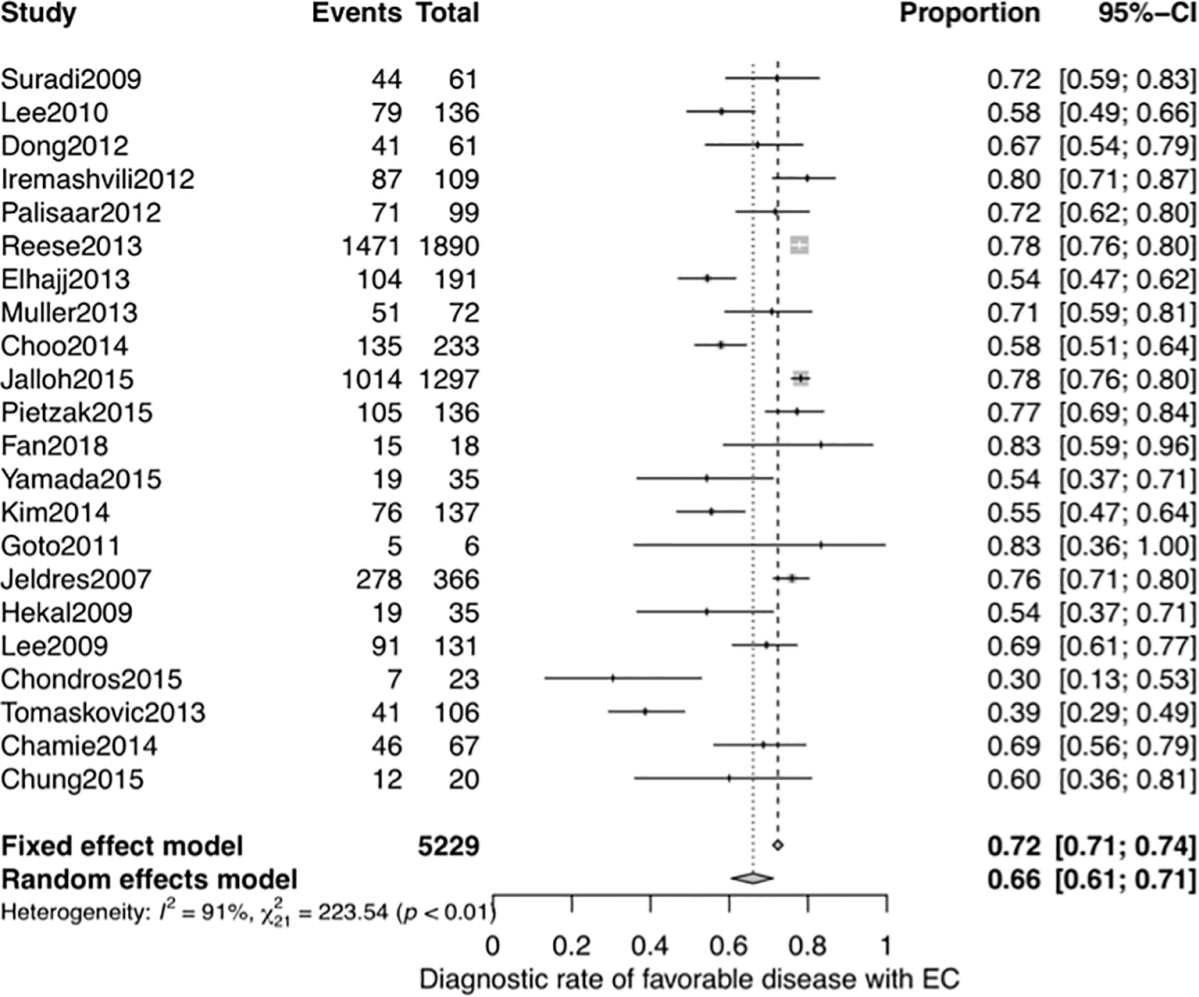
Forest plot of studies that explored the diagnostic accuracy of the EC for favorable disease.

Next, we performed another systematic meta-analysis to validate the diagnostic accuracy of EC in favorable disease. This yielded a total of 5,229 men from 22 studies (4 studies were same to the NMA and 18 studies were additional) (28, 30, 40, 42–54), and a pooled DOR of 0.66 (95% CI, 0.61–0.71, see **Figure 6**). There was also significant heterogeneity in this meta-analysis (I^2^ = 91%). The subgroup analysis of sample size and meta-regression of region (studies performed inside or outside the USA) produced significant results (see **Supplementary Table 4**): the p-values for sample size and region were 0.049 and 0.013, respectively. The pooled DOR of the EC was significantly higher in studies performed in the USA than in those performed in other regions (0.73 vs 0.62, p = 0.013; see **Supplementary Figure 14**). While the significant relationship between sample size and the DOR indicated potential publication bias, the funnel plot for publication bias showed a certain degree of symmetry (see **Supplementary Figure 15**), and the p-value of an Egger’s regression test for plot symmetry was 0.7585. No evidence of publication bias was found.

## Discussion

Identifying patients with purely low-grade prostate cancer is currently problematic because of disease misclassification. The true misclassification rate in these patients is controversial, and the diagnostic abilities of contemporary AS criteria may be overestimated. The diagnostic accuracy of AS criteria can be validated using studies that evaluated pathological outcomes at RP in men who fulfilled AS selection criteria but underwent definitive treatment.

The results of the NMA showed that EC had the best predictive ability for insigPCa, except for the Yonsei criteria, which was evaluated in only 1 study, and sensitivity analysis showed that the results of the NMA were robust regardless of whether a classical or updated definition of insigPCa was used (see **Supplementary Figure 9**). The pooled diagnostic accuracy of the EC for insigPCa was 0.44, indicating that more than half of the cases of prostate cancer that were initially considered clinically “insignificant” were not in fact insignificant. According to the results obtained in previous large AS cohorts, the rate of upgrading at the first repeat biopsy was approximately 30% (14, 55), which is lower than the DOR found for insigPCa using the AS criteria evaluated in this study. Therefore, a separate meta-analysis was performed to validate the DOR of insigPCa using the EC with a more liberal endpoint, favorable disease, which rules out the volume of PCa, which is a restrictive condition. The pooled DOR of the EC for favorable disease was 0.66, which is more consistent with the real-life experience reported in previous large cohorts.

In 2018, the American Urological Association/American Society for Radiation Oncology/Society of Urologic Oncology (AUA/ASTRO/SUO) guidelines announced that given the increase in the number of cores obtained in a systematic biopsy, the definition for a diagnosis of very low-risk PCa should be updated to refer to cases in which no more than 33% of the total cores are positive (instead of those in which no more than two cores are positive, as was stated in the previous version) (56). **Table 1** shows that although the total number of cores obtained during biopsy was more than the traditional six cores in all of the included studies, they all still used “no more than 2 cores” as an eligibility characteristic when applying the EC to diagnose insigPCa. This method may have led to an overestimation of the diagnostic accuracy of the EC, and the true value could therefore be even worse than would be expected based on our results.

Because diagnostic accuracy of criteria designed to identify insigPCa is limited when only a single biopsy is obtained, confirmatory biopsy is recommended as a mandatory step before AS strategy is determined (57). Recently, the ASIST study demonstrated that performing an additional baseline MRI before confirmatory biopsy significantly reduced the rate of upgrading in surveillance biopsies (58). In recent years, multiparametric MRI (mpMRI) has been applied to optimize patient selection and monitoring in AS (59–63). MRI-targeted biopsy showed that confirmatory biopsy did provide additional value in detecting suspicious lesions (64). However, the latest European Urologic Association (EUA) guidelines recommend that men eligible for AS who were diagnosed based on combined systematic and MRI-targeted biopsy do not need a confirmatory biopsy (65). The Prostate Imaging Reporting and Data System (PI-RADS) was established in 2012 (66) and updated in 2015 (v2) (67) and again more recently (v2.1) (68). This approach has minimized the heterogeneity in DOR among different institutions and provided useful supplementary information that may be helpful in preventing incorrect assignment as AS (47, 69). Novel biomarkers, such as PCA3, also urgently need to be incorporated into AS criteria to improve diagnostic accuracy.

Meta-analyses of proportions tend to possess significant heterogeneity (70, 71), and high heterogeneity was also found in the meta-analysis of the individual DOR in this study. We identified the region the study was conducted in (inside or outside the US), the median duration of the study recruitment period, sample size and central review of pathology as potential sources of this heterogeneity. Even so, no publication bias was found in either the NMA or the subsequent meta-analysis of the DOR of the EC.

Institutions in the USA tended to have higher DOR for both insigPCa (0.54 vs 0.40, P-value = 0.32) and favorable disease (0.73 vs 0.62, P-value = 0.013) than was found in those in other regions. Due to a lack of sufficient data, we were unable to further validate the differences between subgroups divided by region. We speculate that the standard measurement of prostate volume (PV) and the use of digital rectal examination (DRE) for clinical T stage in the USA may contribute to the better performance of those institutions. PV was determined by a variety of methods in the included studies, including transrectal ultrasonography (TRUS), MRI, and CT scan or estimations based on RP specimens using different formulas (e.g., length × width × height × 0.52, tumor area × thickness of specimen × 1.1, weight or weight/1.1), and the PV measurements are known to vary considerably according to the method used (72). Indeed, significant inter-observer variation has been identified in PV measurements obtained with TRUS, and DRE used for PCa clinical staging (73, 74). A detailed and standard operating procedure illustration for DRE and PV measurements in the diagnosis of insigPCa are needed to standardize the selection criteria.

Central pathology review would exclude interobserver variability and eliminate variation in the use of the Gleason score system, potentially improving the quality of the study—as such, we set central pathology review (yes or no) as a potential contributor to heterogeneity. However, no significant outcome was detected for insigPCa or favorable disease (see **Supplementary Figures 12**, **16**). It has also been reported that after the 2005 ISUP modification of the Gleason grading system was introduced, the accuracy of the EC in predicting insigPCa declined (75). In an attempt to validate this decline, we further explored the effect of the median study recruitment duration on the DOR as both a dummy variable (before or after 2005) and a continuous variable, and the results showed there were no significant differences in any variable types or endpoints.

To the best of our knowledge, this is the first NMA to pool contemporary AS criteria together to assess their diagnostic accuracies for insigPCa. While our findings should provide both urologists and AS candidates with valuable information, the present study does have some limitations. First and foremost, we extracted only the positive predictive value (PPV) of each AS criteria; because of our limited access to original data, we could not evaluate negative predictive value, specificity or sensitivity; hence, further studies that evaluate insignificant/significant PCa diagnosed based on any AS criteria are needed. Second, because the number of comparative arms was excessive (≥5) in some of the studies included in the NMA, we were unable to perform a heterogeneity analysis of the NMA. Hence, the heterogeneity assessment of the NMA was conducted using a pairwise meta-analysis, revealing moderate heterogeneity. Third, high heterogeneity was found in the meta-analysis of the DOR; however, subgroup and meta-regression analyses found few factors that could explain the heterogeneity. Forth, limited by available studies, the favourable disease was only used as endpoint in the meta-analysis of DOR of EC.

## Conclusion

Among the seven contemporary AS criteria evaluated in this study, the EC performed best in positively selecting patients with insigPCa. While the pooled diagnostic accuracy of the EC for the endpoint insigPCa was 0.44, DOR increased to 0.66 when a more liberal endpoint, favorable disease, was used. High heterogeneity was detected in the analysis of individual AS criteria, and subgroup analysis showed that when the EC was used, institutions located in the USA achieved better diagnostic performance than was found for those located in other regions. A further detailed standard operating procedure of screening criteria application in AS is needed in worldwide practice.

## Data Availability Statement

The original contributions presented in the study are included in the article/**Supplementary Material**. Further inquiries can be directed to the corresponding authors.

## Author Contributions

①YF: Concepulation, Methodology, Software, Validation, Writing-Review & Editing, Supervision, Project administration, Funding acquisition; ②YM: Concepulation, Methodology, Software, Investigation, Formal analysis, Writing-original draft, Review & Editing, Visualization; ③LZ: Concepulation, Investigation; ④YC: Methodology, Resources; ⑤YW: Methodology, Resources; ⑥JF: Concepulation, Methodology; ⑦WY: Concepulation, Funding acquisition; ⑧QZ: Supervision, Project administration, Funding acquisition. All authors contributed to the article and approved the submitted version.

## Funding

This study was supported by grants from the Tibetan Natural Science Foundation of the Medical Group Supporting Program to YF (Grant No. XZ2019ZR-ZY16(Z)) and from the National Natural Science Foundation of China to WY (http://www.nsfc.gov.cn/publish/portal1/) (Grant No. 81870518) and QZ (Grant No. 81872088).

## Conflict of Interest

The authors declare that the research was conducted in the absence of any commercial or financial relationships that could be construed as a potential conflict of interest.

## Publisher’s Note

All claims expressed in this article are solely those of the authors and do not necessarily represent those of their affiliated organizations, or those of the publisher, the editors and the reviewers. Any product that may be evaluated in this article, or claim that may be made by its manufacturer, is not guaranteed or endorsed by the publisher.
