# Supplementary material for: Diagnostic Accuracy of Contemporary Selection Criteria in Prostate Cancer Patients Eligible for Active Surveillance: A Bayesian Network Meta-Analysis
Source: Front Oncol. 2022 Jan 10;11:810736. doi: 10.3389/fonc.2021.810736 (PMC8785217; doi:10.3389/fonc.2021.810736)
Supplement: Supplementary file 1 [file Table_1.docx]

Supplementary Table 1. Active surveillance criteria identified in the network analysis.

| AS criteria | Biopsy Gleason Score | Clinical Tumor Stage | PSA (ng/ml) | PSA Density (ng/ml/cc) | Positive Biopsy Cores, n | Maximum Cancer per Core, % |
| --- | --- | --- | --- | --- | --- | --- |
| EC | ≤3+3 | T1c | - | ≤0.15 | ≤2 (≤33% total cores, updated in 2018) | ≤50 |
| Yonsei | ≤3+3 | T1c-T2c | ≤10 | - | ≤1 | ≤50 |
| PRIAS | ≤3+3 | T1c-T2c | ≤10 | ≤0.20 | ≤2 | - |
| MSKCC | ≤3+3 | T1c-T2a | ≤10 | - | ≤2 | ≤50 |
| UM | ≤3+3 | T1c-T2c | ≤10 | - | ≤2 | ≤20 |
| UCSF | ≤3+3 | T1c-T2a | ≤10 | - | ≤33% (at least 6) | - |
| UT | ≤3+3 | T1c-T2c | ≤10 | - | ≤2 | - |

Note: EC= Epstein Criteria, PRIAS= Prostate Cancer Research International: Active Surveillance, MSKCC= Memorial Sloan-Kettering Cancer Center, UCSF= University of California, San Francisco, UM= University of Miami, UT= University of Toronto.

Supplementary Table 2. Diagnostic accuracy of each AS criteria to identify patients with pathologically insignificant prostate cancer with different definitions.

| **Study** | **Year** | **Included AS protocols** | **No. of patients eligible to AS** | **No. of insigPCa**  **classical def.[n (%)]** | **No. of insigPCa** **updated def. [n (%)]** |
| --- | --- | --- | --- | --- | --- |
| Cantiello et al^[16]^ | 2015 | PRIAS | 188 | NA | 115 (0.61) |
|  |  | EC | 96 | NA | 61 (0.64) |
| Iremashvili et al^[17]^ | 2012 | EC | 109 | 65 (0.60) | 85 (0.78) |
|  |  | MSKCC | 246 | 115 (0.47) | 171 (0.70) |
|  |  | PRIAS | 190 | 103 (0.54) | 142 (0.75) |
|  |  | UCSF | 270 | 121 (0.45) | 181 (0.67) |
|  |  | UM | 189 | 104 (0.55) | 138 (0.73) |
| Kang et al^[18]^ | 2015 | EC | 70 | 55 (0.79) | NA |
|  |  | MSKCC | 161 | 119 (0.74) | NA |
|  |  | PRIAS | 109 | 79 (0.72) | NA |
|  |  | UCSF | 141 | 104 (0.74) | NA |
|  |  | UM | 96 | 72 (0.75) | NA |
| Kim et al^[19]^ | 2014 | EC | 137 | 22 (0.16) | NA |
|  |  | UT | 387 | 35 (0.09) | NA |
|  |  | UCSF | 334 | 34 (0.10) | NA |
|  |  | PRIAS | 226 | 32 (0.14) | NA |
|  |  | UM | 222 | 29 (0.13) | NA |
|  |  | MSKCC | 322 | 34 (0.11) | NA |
| Lim et al^[20]^ | 2013 | EC | 31 | 10 (0.32) | NA |
|  |  | MSKCC | 121 | 25 (0.21) | NA |
|  |  | PRIAS | 101 | 23 (0.23) | NA |
|  |  | UCSF | 159 | 29 (0.18) | NA |
|  |  | UM | 88 | 20 (0.23) | NA |
|  |  | Yonsei | 69 | 17 (0.25) | NA |
| Palisaar et al^[21]^ | 2012 | MSKCC | 308 | 66 (0.21) | NA |
|  |  | EC | 99 | 28 (0.28) | NA |
|  |  | UT | 514 | 59 (0.11) | NA |
|  |  | PRIAS | 174 | 41 (0.24) | NA |
| Yamada et al^[22]^ | 2015 | EC | 35 | 14 (0.40) | 18 (0.51) |
|  |  | PRIAS | 55 | 18 (0.33) | 23 (0.42) |
|  |  | UM | 69 | 19 (0.28) | 27 (0.39) |
|  |  | UCSF | 89 | 21 (0.24) | 29 (0.33) |
|  |  | MSKCC | 92 | 21 (0.23) | 30 (0.33) |
|  |  | UT | 118 | 22 (0.19) | 32 (0.27) |

Note: insigPCa=insignificant prostate cancer, EC= Epstein Criteria, PRIAS= Prostate Cancer Research International: Active Surveillance, MSKCC= Memorial Sloan-Kettering Cancer Center, UCSF= University of California, San Francisco, UM= University of Miami, UT=University of Toronto.

Supplementary Table 3. Subgroup analysis and meta-regression of diagnostic rate (endpoint: insigPCa ).

| Subgroups/Variables | Estimate | S.E. | P-value |
| --- | --- | --- | --- |
| Region (USA or Others) | 0.5834 | 0.5918 | 0.3242 |
| Median year of study recruiting | 0.0843 | 0.0845 | 0.3186 |
| Sample size | 0.0013 | 0.0041 | 0.7509 |
| Pathology central review (Yes or No) | -0.0795 | 0.6185 | 0.8978 |

Supplementary Table 4. Subgroup analysis and meta-regression of diagnostic rate (endpoint: favorable disease).

| Subgroups/Variables | Estimate | S.E. | P-value |
| --- | --- | --- | --- |
| Region (USA or Others) | 0.5087 | 0.2044 | 0.0128* |
| Median year of study recruiting | -0.0504 | 0.0411 | 0.2200 |
| Sample size | 0.0005 | 0.0002 | 0.0490* |
| Pathology central review (Yes or No) | -0.0417 | 0.2386 | 0.8612 |

Note: *represents a significant outcome.
