# Supplementary figures and images for: Diagnostic Accuracy of Contemporary Selection Criteria in Prostate Cancer Patients Eligible for Active Surveillance: A Bayesian Network Meta-Analysis
Source: Front Oncol. 2022 Jan 10;11:810736. doi: 10.3389/fonc.2021.810736 (PMC8785217; doi:10.3389/fonc.2021.810736)

## Slide 1
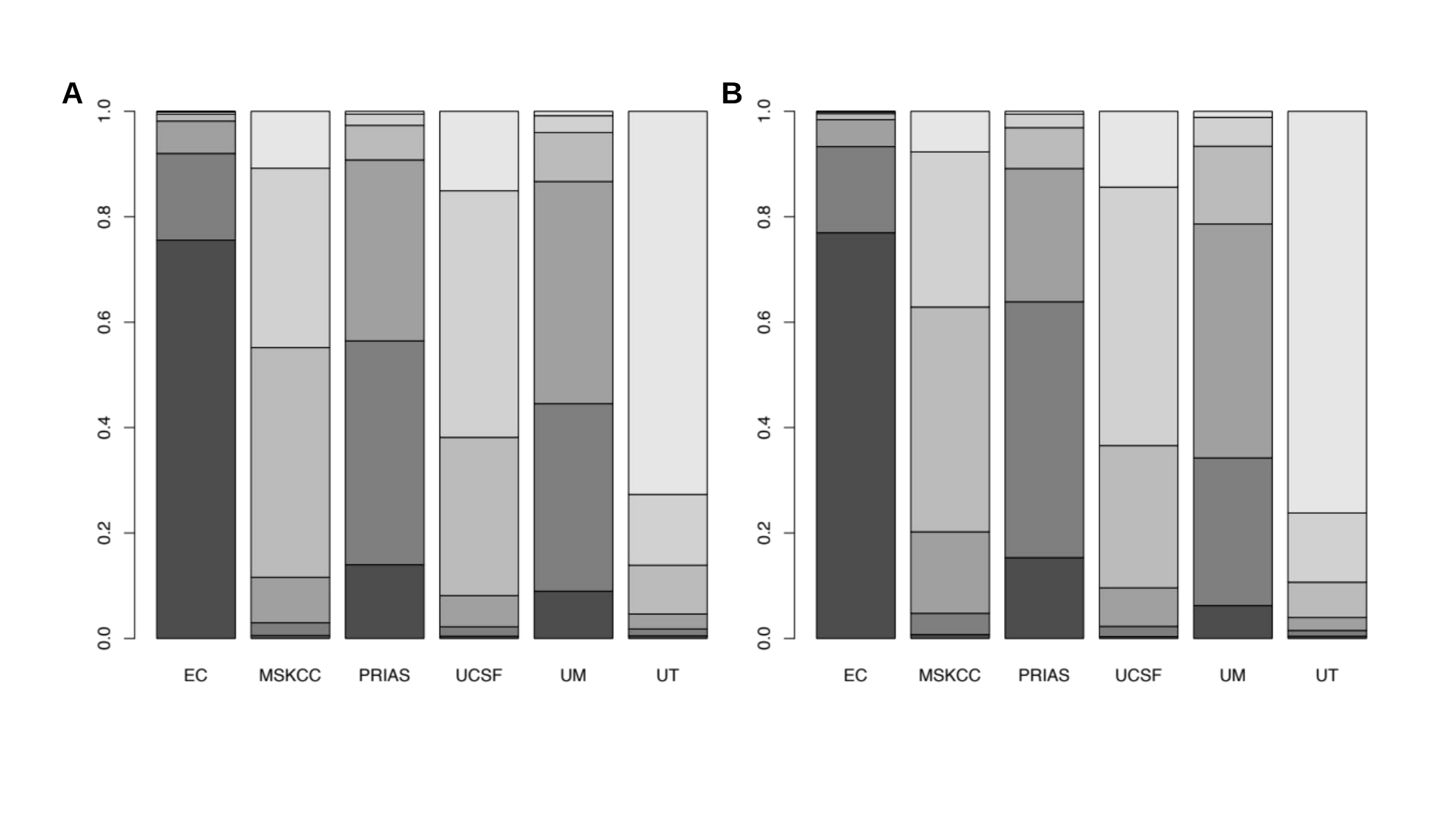

A
B

Supplement: Supplementary file 2 [file Presentation_1.pptx]

## Slide 1
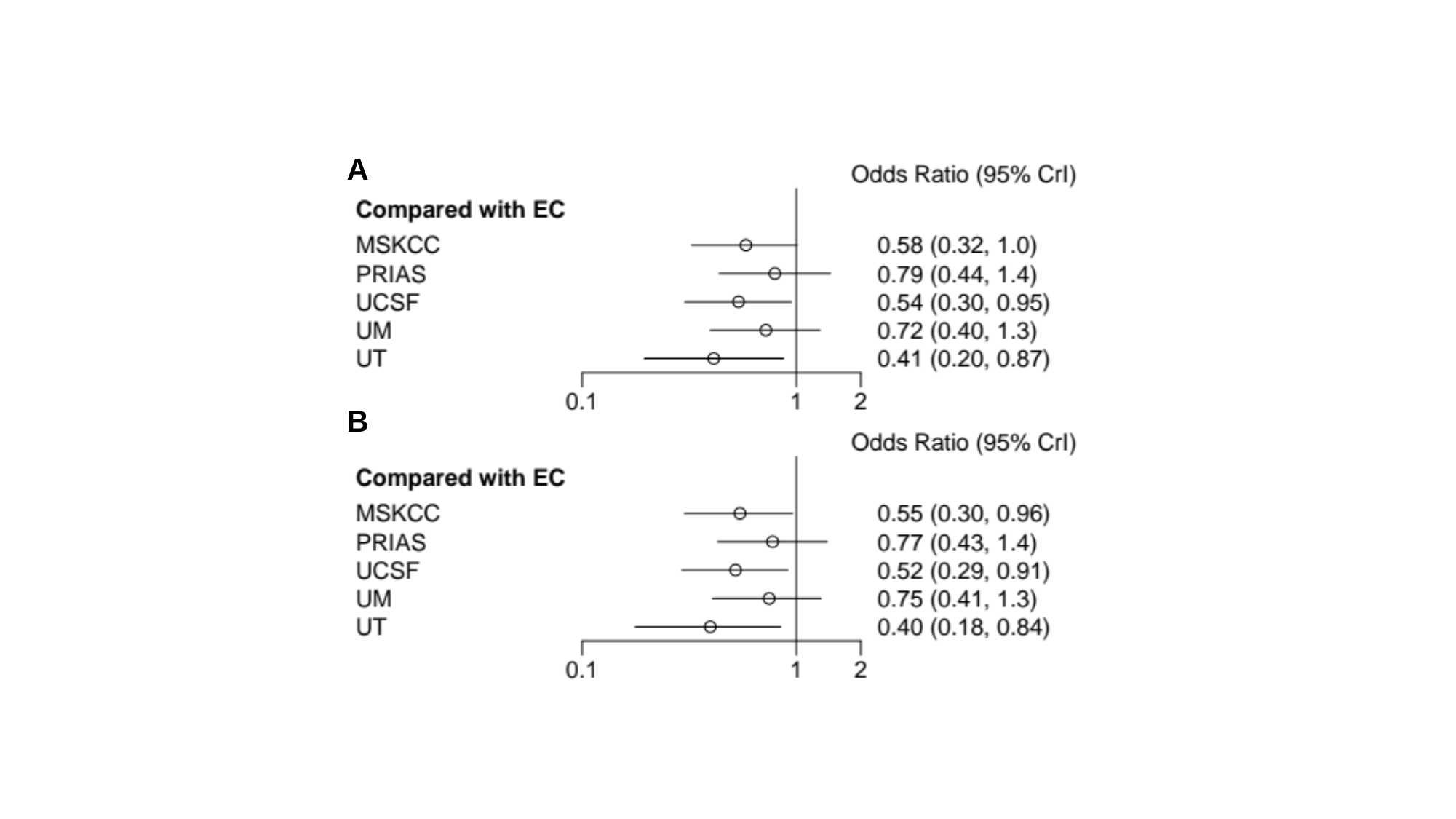

A
B

Supplement: Supplementary file 3 [file Presentation_2.pptx]

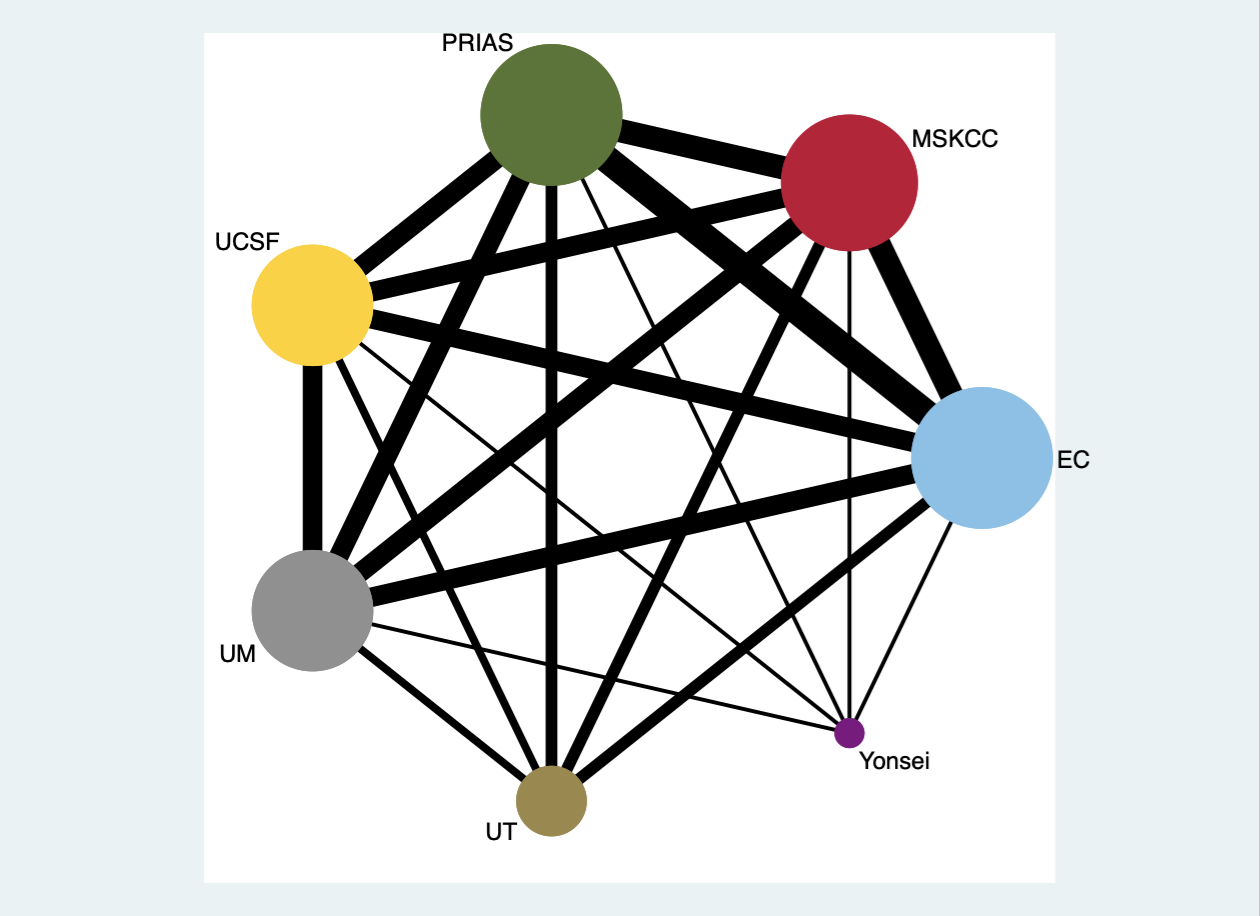

Supplement: Supplementary file 4 [file Image_1.tif]

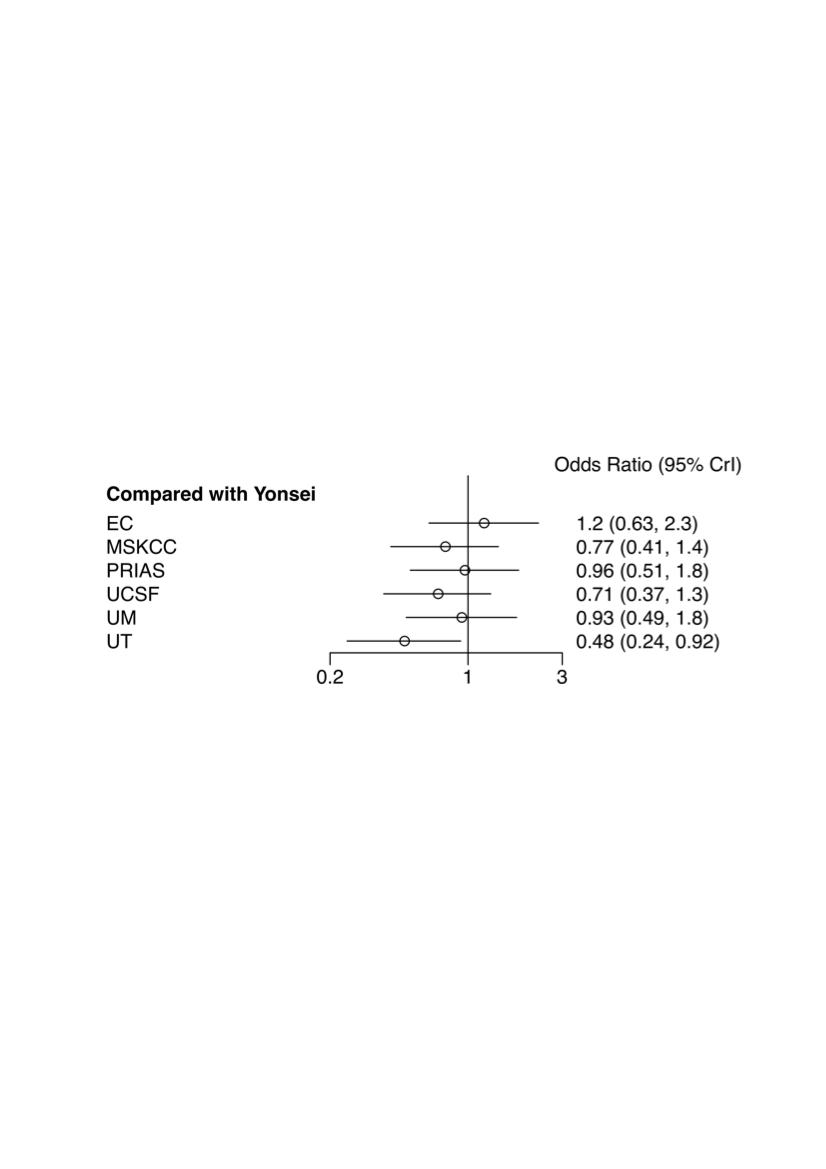

Supplement: Supplementary file 5 [file Image_2.tiff]

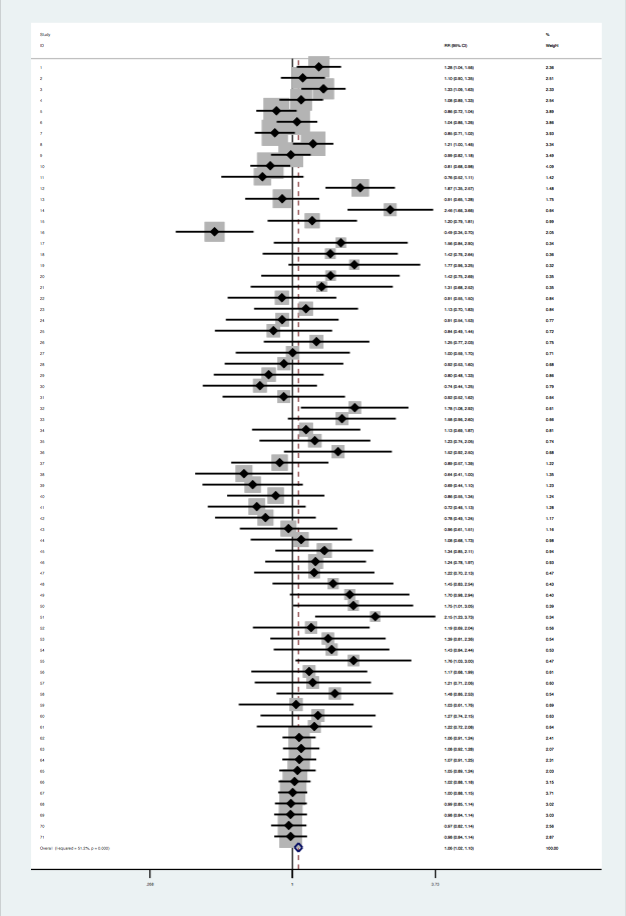

Supplement: Supplementary file 6 [file Image_3.tif]

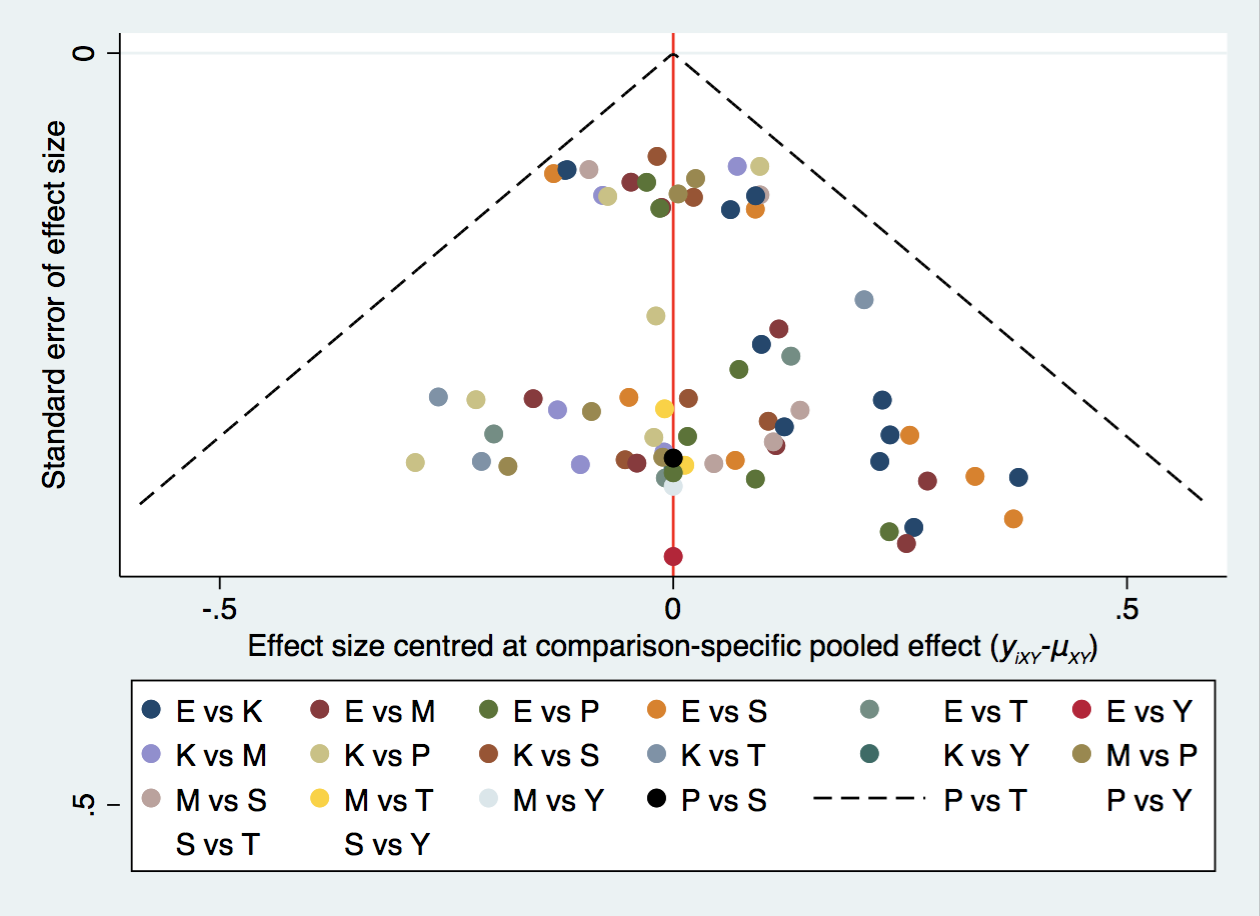

Supplement: Supplementary file 7 [file Image_4.tif]

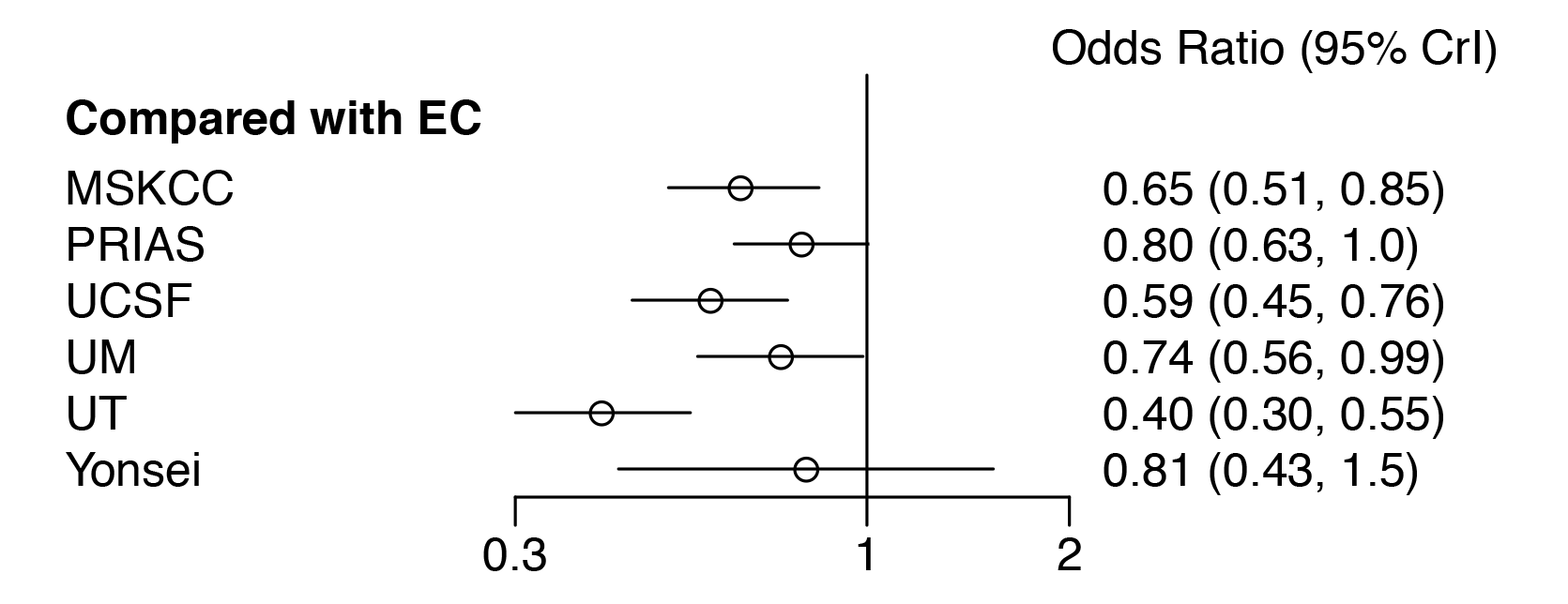

Supplement: Supplementary file 8 [file Image_5.tif]

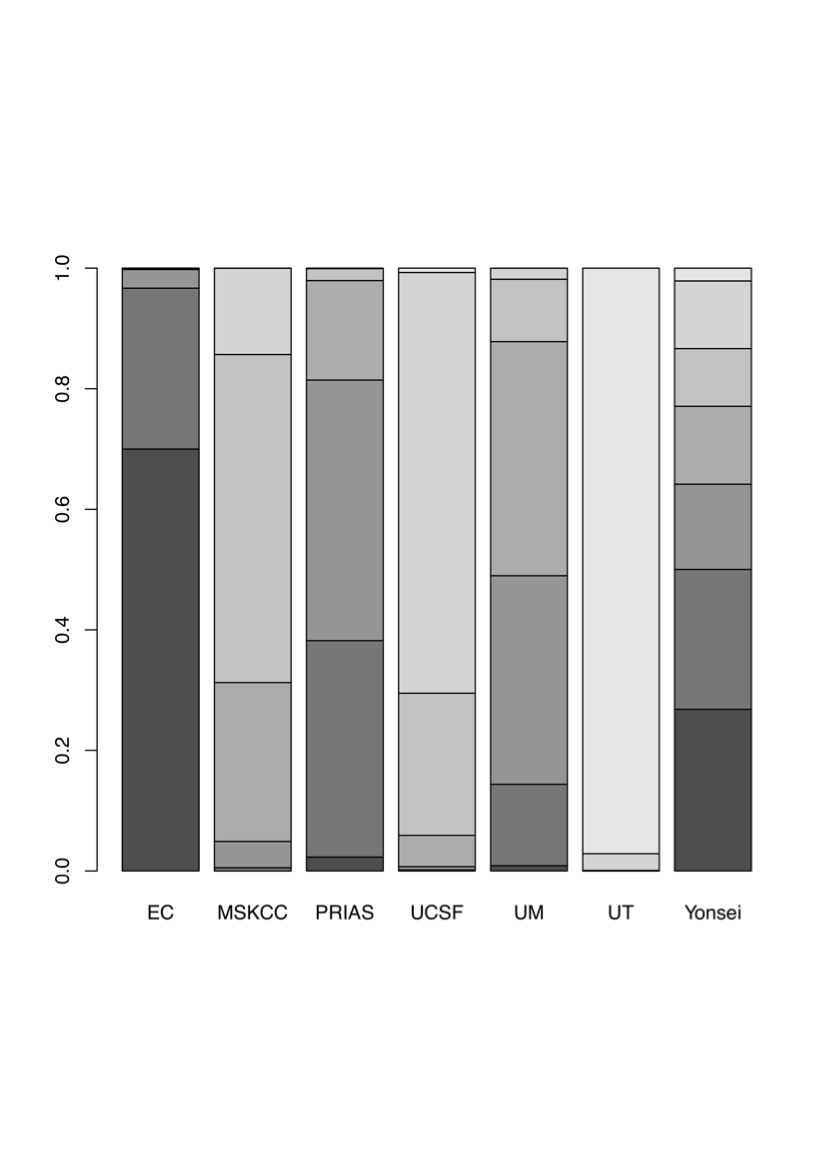

Supplement: Supplementary file 9 [file Image_6.tiff]

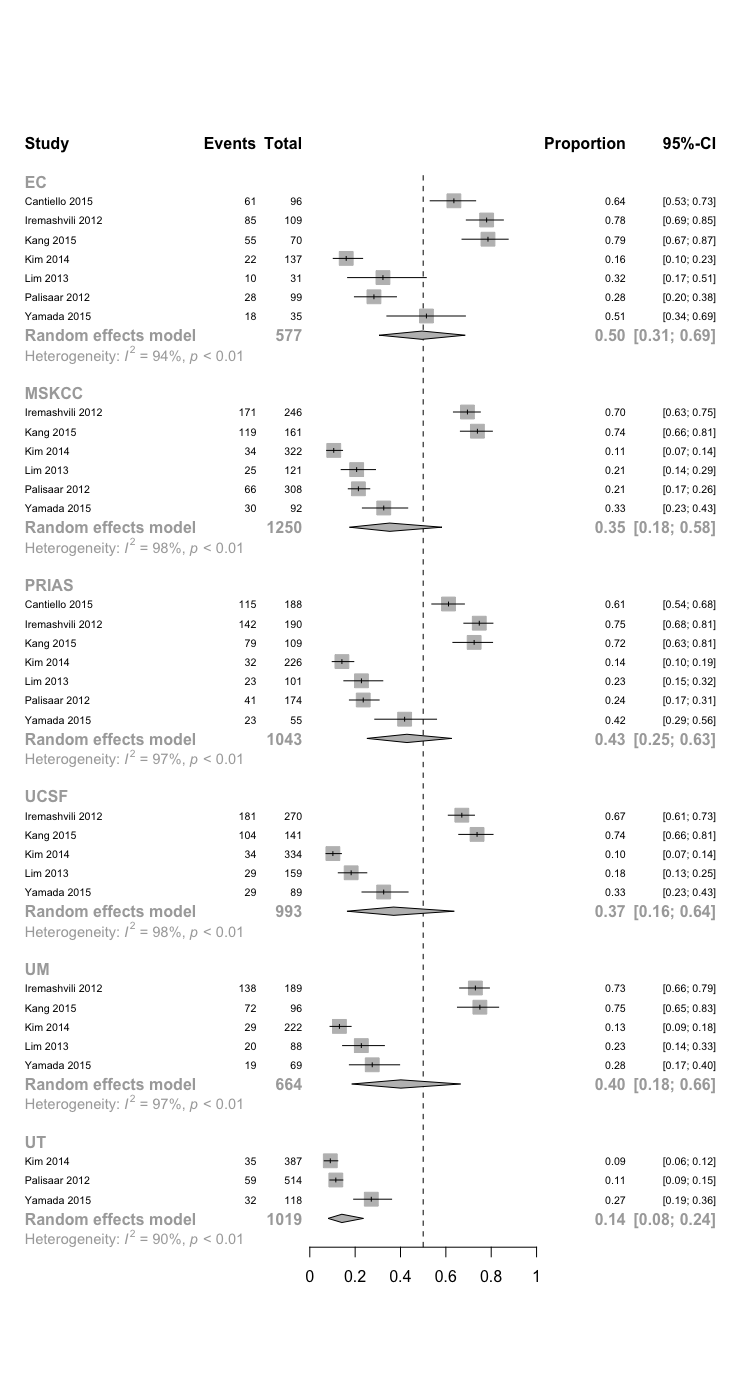

Supplement: Supplementary file 10 [file Image_7.tif]

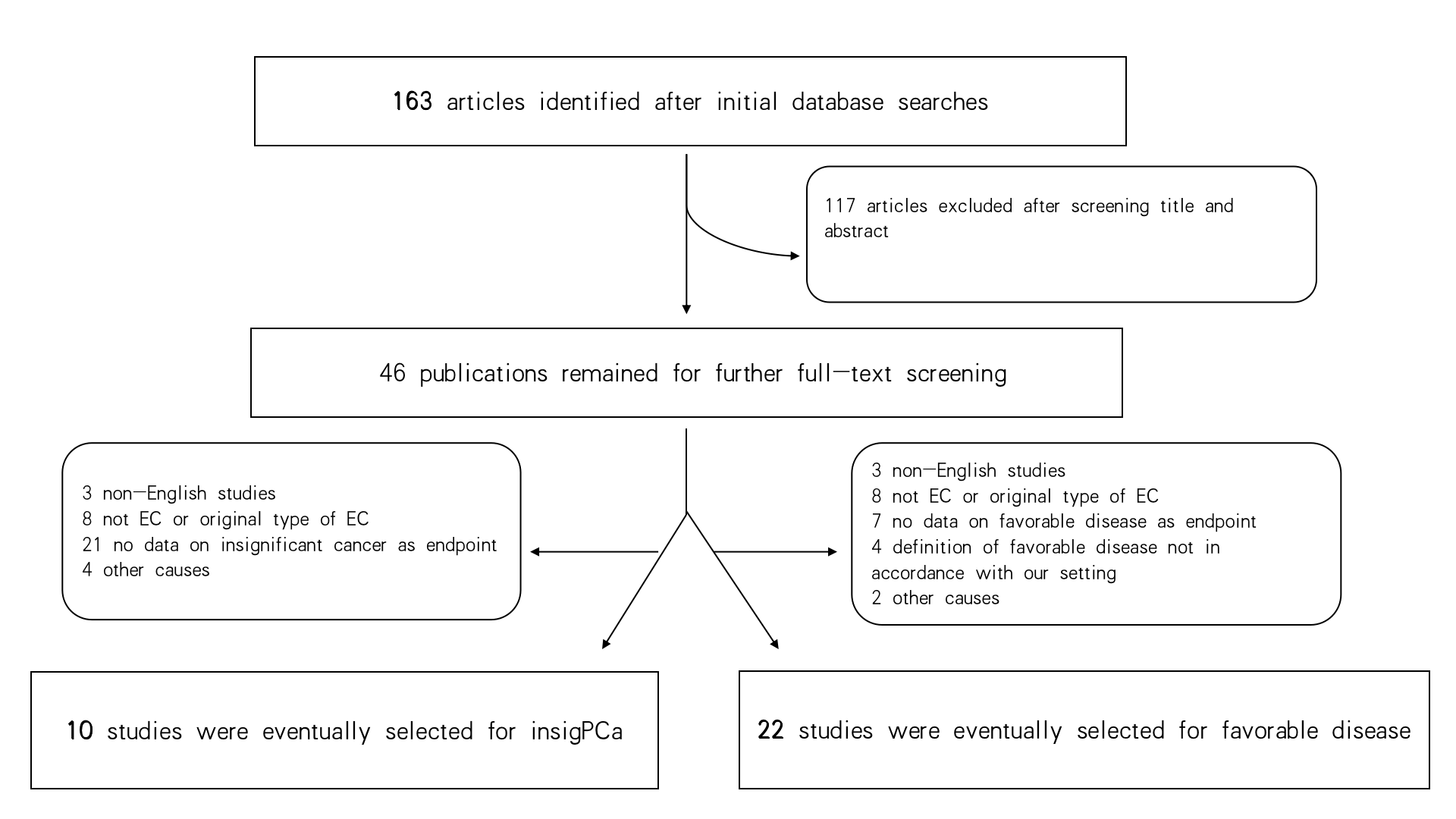

Supplement: Supplementary file 11 [file Image_8.tif]

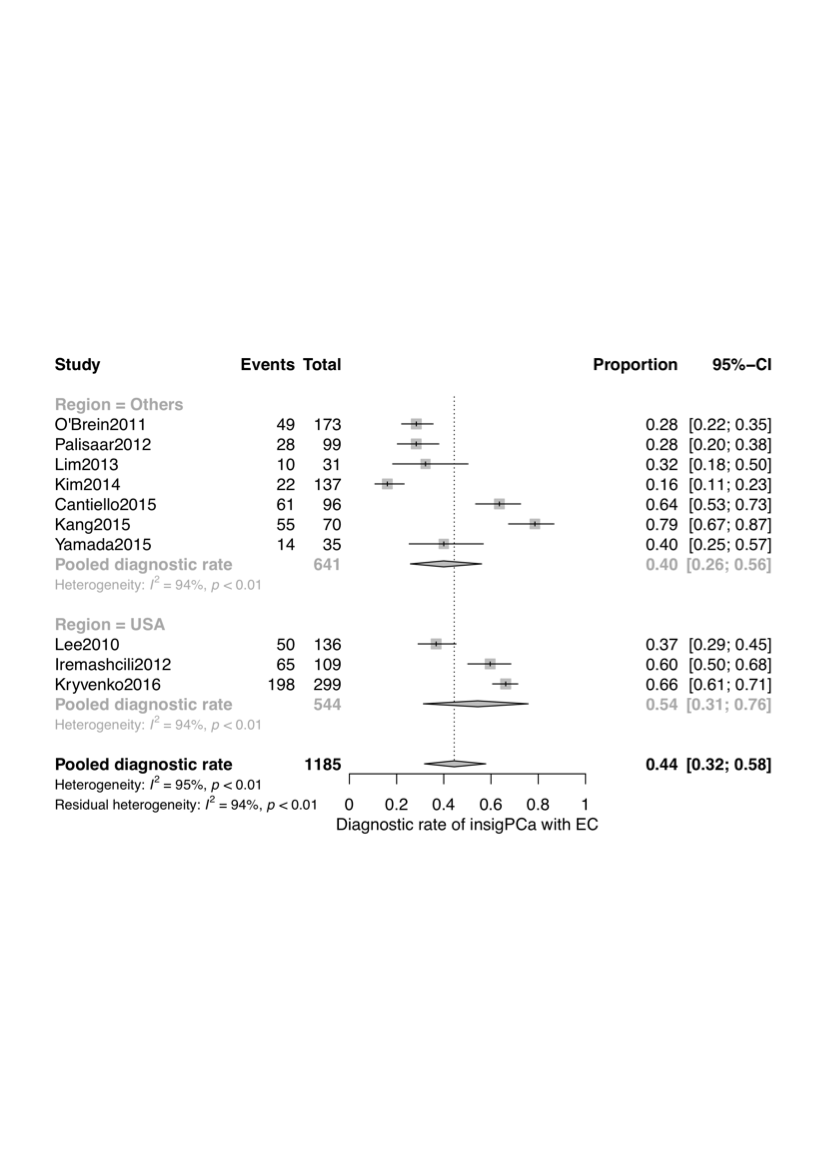

Supplement: Supplementary file 12 [file Image_9.tiff]

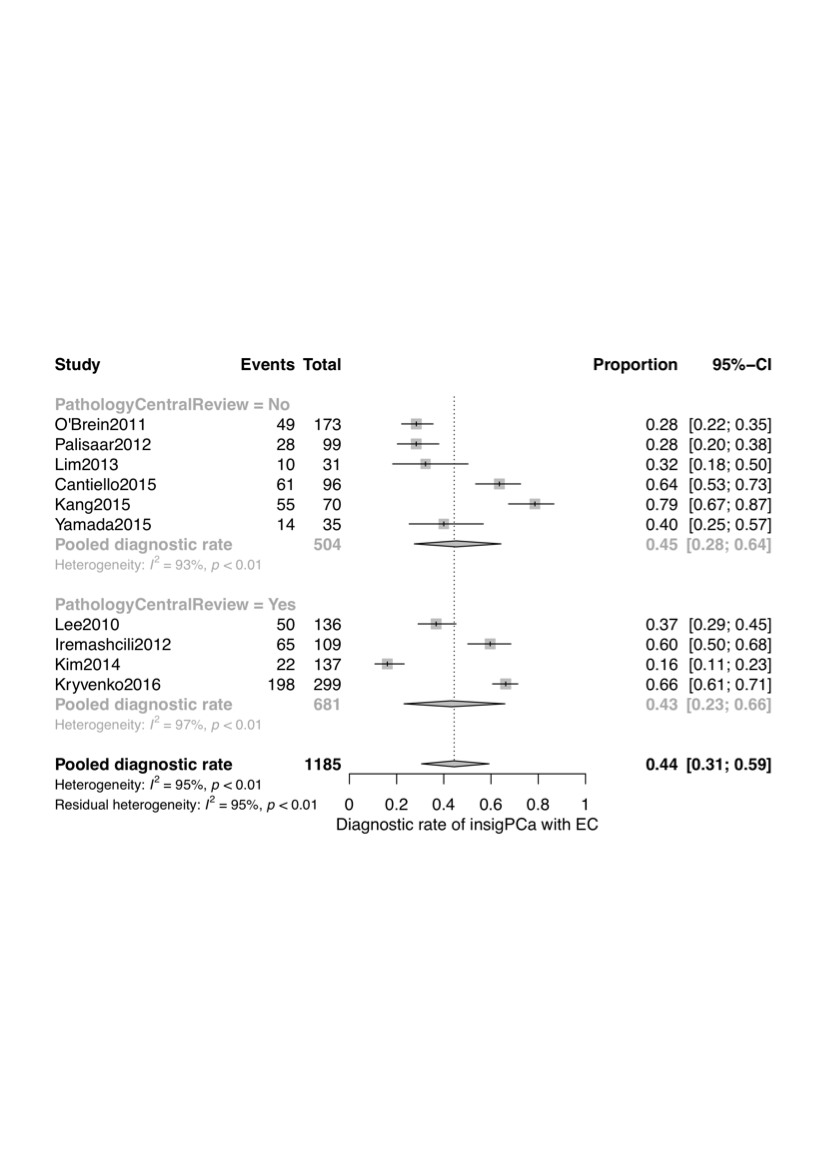

Supplement: Supplementary file 13 [file Image_10.tiff]

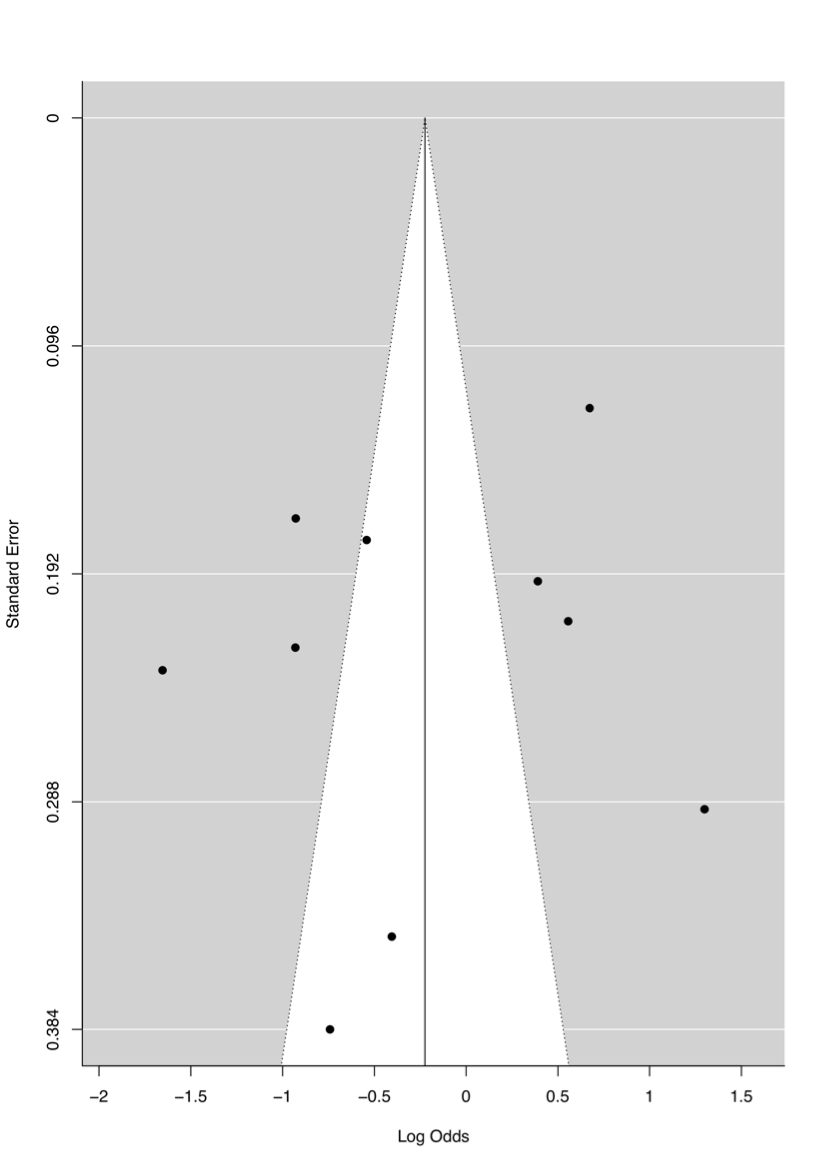

Supplement: Supplementary file 14 [file Image_11.tiff]

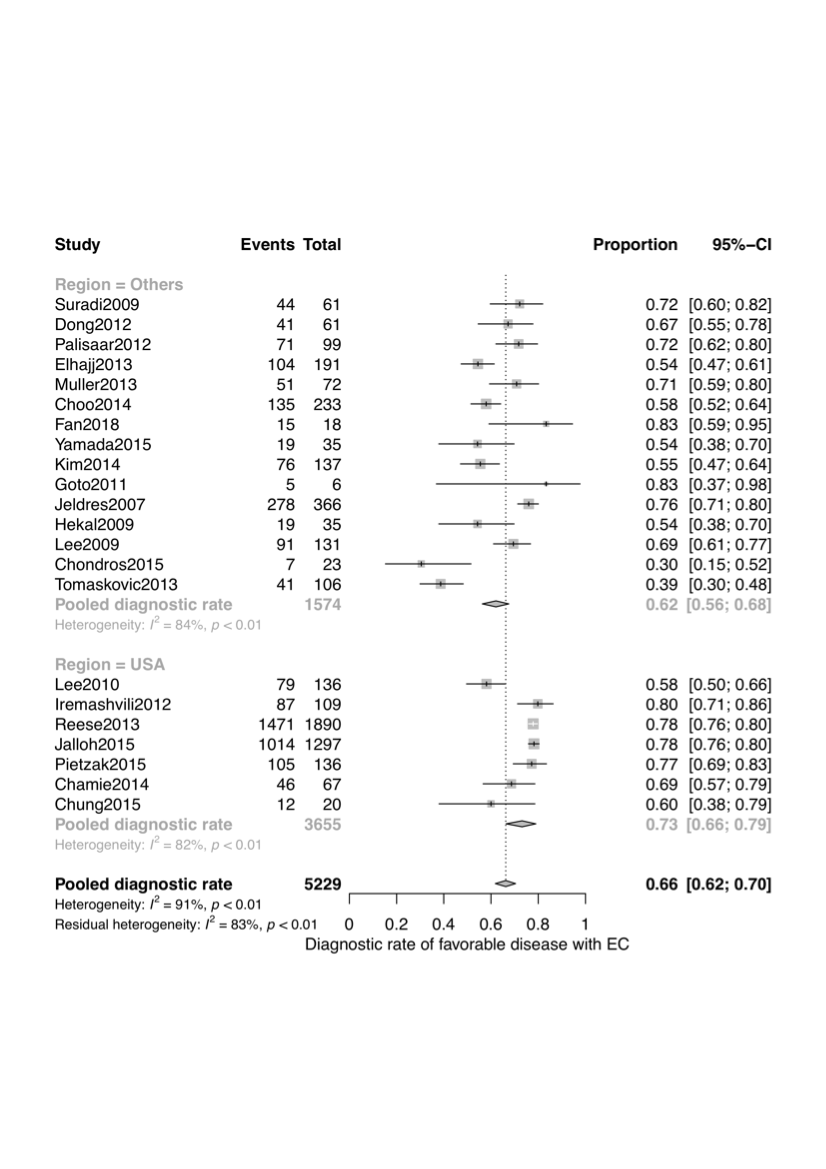

Supplement: Supplementary file 15 [file Image_12.tiff]

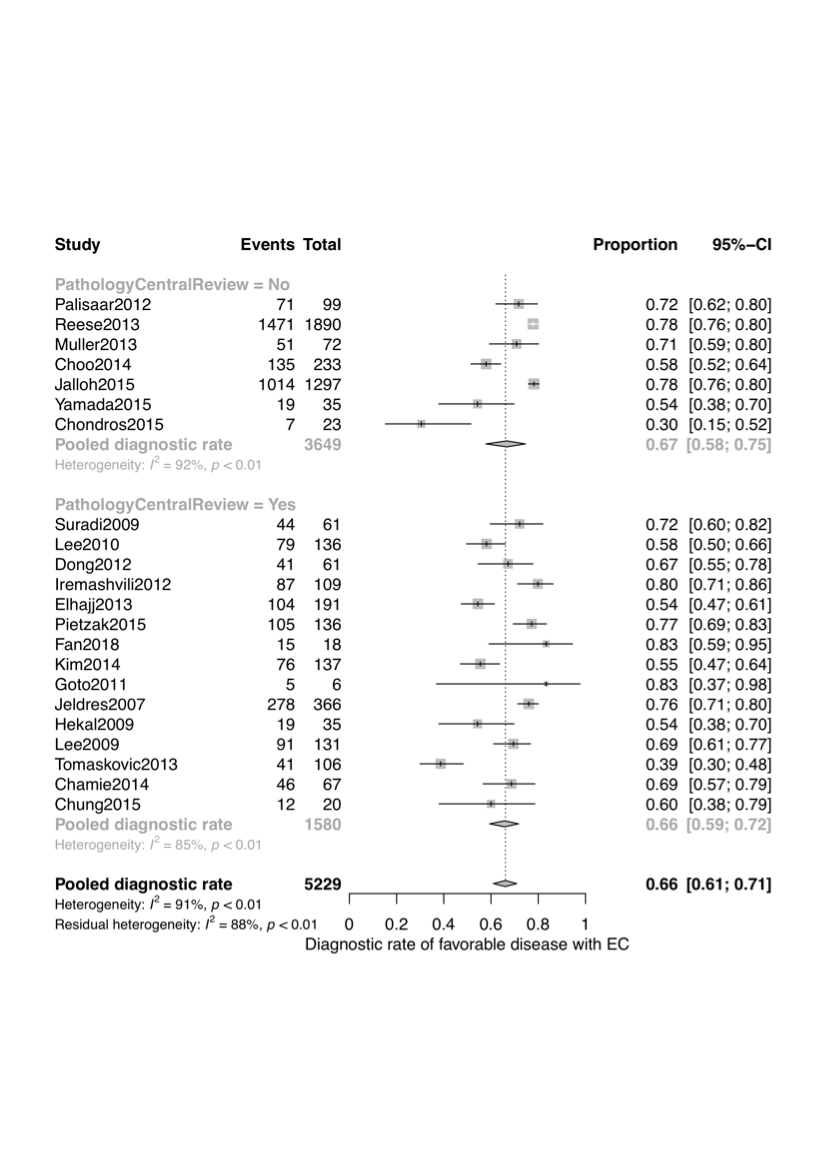

Supplement: Supplementary file 16 [file Image_13.tiff]

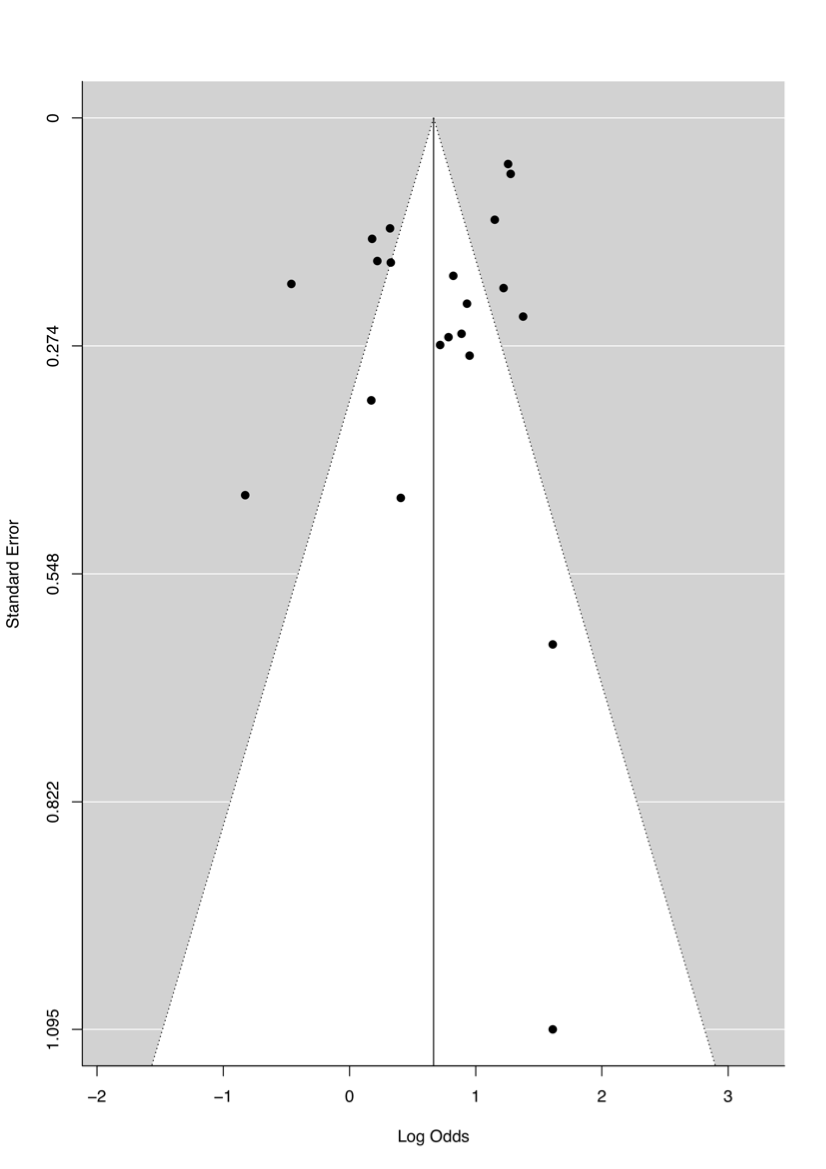

Supplement: Supplementary file 17 [file Image_14.tiff]
